# Parental Access to Children's Raw Genomic Data in Canada: Legal Rights and Professional Responsibility

**DOI:** 10.3389/fgene.2021.535340

**Published:** 2021-03-31

**Authors:** Michael J. S. Beauvais, Adrian M. Thorogood, Michael J. Szego, Karine Sénécal, Ma'n H. Zawati, Bartha Maria Knoppers

**Affiliations:** ^1^Centre of Genomics and Policy, Faculty of Medicine, McGill University, Montreal, QC, Canada; ^2^ELIXIR-LU, Luxembourg Centre for Systems Biomedicine, University of Luxembourg, Belvaux, Luxembourg; ^3^Centre for Clinical Ethics, Unity Health, Toronto, ON, Canada; ^4^Departments of Family and Community Medicine and Molecular Genetics, Dalla Lana School of Public Health, University of Toronto, Toronto, ON, Canada; ^5^Independent Researcher, Montreal, QC, Canada

**Keywords:** whole genome sequencing, personal genomics, pediatrics, privacy, individual access, ethics, consent

## Abstract

Children with rare and common diseases now undergo whole genome sequencing (WGS) in clinical and research contexts. Parents sometimes request access to their child's raw genomic data, to pursue their own analyses or for onward sharing with health professionals and researchers. These requests raise legal, ethical, and practical issues for professionals and parents alike. The advent of widespread WGS in pediatrics occurs in a context where privacy and data protection law remains focused on giving individuals control-oriented rights with respect to their personal information. Acting in their child's stead and in their best interests, parents are generally the ones who will be exercising these informational rights on behalf of the child. In this paper, we map the contours of parental authority to access their child's raw genomic data. We consider three use cases: hospital-based researchers, healthcare professionals acting in a clinical-diagnostic capacity, and “pure” academic researchers at a public institution. Our research seeks to answer two principal questions: Do parents have a right of access to their child's raw WGS data? If so, what are the limits of this right? Primarily focused on the laws of Ontario, Canada's most populous province, with a secondary focus on Canada's three other most populous provinces (Quebec, British Columbia, and Alberta) and the European Union, our principal findings include (1) parents have a general right of access to information about their children, but that the access right is more capacious in the clinical context than in the research context; (2) the right of access extends to personal data in raw form; (3) a consideration of the best interests of the child may materially limit the legal rights of parents to access data about their child; (4) the ability to exercise rights of access are transferred from parents to children when they gain decision-making capacity in both the clinical and research contexts, but with more nuance in the former. With these findings in mind, we argue that professional guidelines, which are concerned with obligations to interpret and return results, may assist in furthering a child's best interests in the context of legal access rights. We conclude by crafting recommendations for healthcare professionals in the clinical and research contexts when faced with a parental request for a child's raw genomic data.

## Introduction

Children with rare and common diseases now undergo whole genome sequencing (WGS) in clinical and research contexts. Parents sometimes request access to their child's raw genomic data, to pursue their own analyses or for onward sharing with health professionals and researchers. These requests raise legal, ethical, and practical issues for both professionals and parents. In general, WGS provides a complete catalog of each nucleotide within an individual's genome. When analyzed with the appropriate tools and expertise, WGS potentially reveals inherited predispositions to a multiplicity of traits and disorders. WGS may also reveal information about a child's future health, which raises the issue of safeguarding the child's ethical right to an open future (Feinberg, 1980), viz. their ability to decide for themselves as adults whether or not to be tested for certain conditions. In turn, this raises the following questions: Should children be tested for adult-onset conditions? Should secondary or incidental findings from WGS be reported to children?

Parental access appears to be an increasingly pressing question for healthcare institutions, clinicians and researchers. The prevalence of parental access requests has not been well-studied, though there is some preliminary empirical evidence of the prevalence of individual access requests generally (Narayanasamy et al., 2020). Patients and caregivers at pediatric institutions occasionally ask for their raw genomic data following WGS tests. Indeed, geneticists at a large pediatric hospital in Ontario report that these requests occur (personal communication) and one of the authors of this article (MS) has been contacted by clinicians and researchers asking for guidance on how to respond to requests for raw genomic data by parents.

Parents may seek access to their child's genomic data for a number of reasons: to seek a second medical opinion about the child's condition, to inform the parents' health or reproductive choices, to share data with a health research project or repository, or to analyze the data themselves to better understand health conditions affecting their child or entire family (though, importantly, their motivation may be unknown in the context of legal access requests). We expect parental access to become more pressing in coming years as a result of three trends: (1) patients are taking a greater role in directing their care and managing their data^1^, (2) sequencing of children is expanding, particularly to new clinical and newborn screening contexts^2^, and (3) a growing ecosystem of third party interpretation services and data sharing platforms are emerging directed toward patients (Capaci et al., 2020; Guerrini et al., 2020).

For health professionals and researchers, parental access raises concerns that children's data will be misinterpreted or misused by parents or third party services, thus putting at risk the child's current and future health interests, development, autonomy, and privacy. Parents may pursue unnecessary analyses discouraged or prohibited by professional organizations, such as analyzing genomes for predispositions for untreatable, adult-onset conditions or predictive adult-onset disease (Borry et al., 2009; Knoppers et al., 2014; Botkin et al., 2015). This may lead to psychosocial harms for the child (e.g., anxiety, low self-esteem) or affect familial relationships (Kesserwan et al., 2016). Parents might publish a child's genome on an open-access recreational genomic database, share it with various researchers and service providers, or unintentionally allow it to be leaked (Bala, 2020). This poses potential risks of genetic discrimination by employers or insurers, and unfettered searches from law enforcement seeking to identify criminal suspects. Parental access to their child's genetic data adds a new molecular dimension to the larger policy debate over the ethics and regulation of “sharenting,” where parents post photos, videos or comments about their children on social media. “Sharenting” can expose children to risks including discrimination, identity theft, reputational harm, and intimidation (Steinberg, 2016).

Previous literature has addressed issues for adults seeking access to raw WGS data. Individuals in many countries have a general right to access their health information^3^ (Ries, 2010; Ogbogu et al., 2014; European Union, 2016; Guerrini et al., 2019). When WGS is adopted in clinical contexts, this right presumably extends to raw WGS data (Thorogood et al., 2018). It remains to be determined whether access to raw data extends to the research context. Many countries exempt researchers from obligations to provide participants access to their personal information (Thorogood et al., 2018). Genomics, however, often blurs clinic and research contexts, raising uncertainty as to the applicability of these exceptions (Schickhardt et al., 2020). Ethically, some commentators express concern that raw genomic data is incomprehensible to most people, offering limited benefit while presenting health and privacy risks to sequenced individuals or their family members stemming from misinterpretation or mismanagement of data (Bredenoord et al., 2011). These concerns are counteracted by principled arguments, including that such data belongs to the individual, who should be free to decide what to do with the data (Schickhardt et al., 2020), arguments of beneficence that individuals can improve their health through sharing data with clinicians or via self-analysis, and finally, utilitarian arguments that providing access may attract research participants as well as provide them with opportunities to accelerate research by sharing their data with other research projects (Kish and Topol, 2015). There are also practical questions about who will provide individuals requesting access with interpretive support and who will pay for this support.

These legal and ethical debates have largely overlooked the rights of children themselves. The legal access rights of parents and the duties of health professionals toward children must be considered in light of their human rights. The United Nations *Convention on the Rights of the Child* (CRC) mandates that the best interests of children are to be a primary consideration in all matters concerning children (United Nations General Assembly, 2007). Parents are authorized by law to act on behalf of their children, while children have a right to be heard and a right to participate in decisions concerning their health (to the extent possible). At the same time, children have a right to appropriate guidance from their parents, amounting to a zone of deference to parental decision-making (Kamchedzera, 2012). Parental authority, however, has a fiduciary character and must be exercised in the child's best interests, not in the parents' personal interests or those of other family members (Tobin and Varadan, 2019). Health professionals are obliged not only to promote the health of children but also to protect children from parental decisions that are contrary to children's actual and future health and well-being (Schwarz et al., 2015). Medical “neglect” under child protection legislation can include both over-treatment or the failure to prevent or treat diseases in children. Parental access therefore raises new legal and ethical questions. When does parental access to their child's information support the child's best interests? When does it threaten them? And who is ultimately responsible for making this determination?

This article examines the legal rights of parents to access their child's raw WGS data generated in healthcare and health research contexts. We begin with legal questions about the scope of parental authority to request access, the rights of children themselves, and the scope of professional responsibility toward minors to justify withholding access. The analysis looks primarily at the freedom of information and health privacy laws of Ontario, Canada's most populous province and the site of much WGS, but also highlights important similarities and differences with the laws of other Canadian provinces and the European Union. While the specific results of our analysis may be largely jurisdiction-specific, we believe the structure of our analysis can be generalized. More specifically, we aim to answer the following questions:

1. Do parents have a legal right to access their child's health information upon request in clinical and research contexts?
2. Do access rights, where applicable, extend to raw genomic sequence data (e.g., BCL, FASTQ, SAM, BAM, or VCF files)?
3. Under what circumstances, if any, can a healthcare institution or researcher refuse a parental access request (e.g., to protect the child's best interests)?
4. Where a minor is sufficiently mature to understand and appreciate the consequences of access requests, does the legal right of access ultimately reside with the minor?

We then turn to contextualizing these findings within the broader, ongoing discussion in ethical and professional guidelines in pediatric genomics surrounding the reporting of secondary and incidental findings (Jarvik et al., 2014; Knoppers et al., 2014; Zawati et al., 2014; Boycott et al., 2015; Sénécal et al., 2015a; Vears et al., 2018). Professional obligations to report secondary or incidental findings to patients or participants are admittedly distinct from the legal obligation to provide an individual access on request. With secondary or incidental findings, professionals have obligations to interpret and “push” information of clinical significance to individuals. In the access context, patients have informational rights to “pull” information (e.g., raw WGS data) upon request from data custodians. While distinct, debates over reporting secondary and incidental findings suggest that health professionals and researchers responding to parental access requests are confronted with important ethical issues surrounding the child's well-being, privacy, and developing autonomy.

Ultimately, our legal analysis aims to guide health researchers, clinicians, and health-care organizations confronted with formal requests from parents to access their child's raw genomic sequence data. In contributing to a better understanding of the law, our research findings can inform access policy and communication between health professionals, parents, and children to ensure the child's health, privacy, and developing autonomy are given full consideration.

## Whole Genome Sequencing in Clinical and Research Contexts

Over the past 35 years, scientists have discovered and studied genetic variants involved in monogenic diseases, resulting in the development of genetic tests for the diagnosis or prediction of monogenic diseases. Advances in molecular biology and other biotechnological advances have contributed to a rapid increase in the supply of genetic tests for hereditary diseases. Since around 2010, next-generation sequencing (NGS) technologies, of which whole genome sequencing (WGS) is a part, have been an important addition to existing genetic testing strategies (Hall et al., 2014). Introduced first in the research realm, they have also had a tremendous impact in the clinical context (Brown and Meloche, 2016; Vaxillaire and Froguel, 2016). For example, the use of WGS presents the possibility of identifying the causes of variable clinical responses among patients with the same condition (Eckford et al., 2019).

Pediatrics has seen some of the first clinical applications of genomics. Genomic sequencing allows for faster diagnosis of inherited and de novo disease and increases the likelihood of diagnosing a child with a rare disease, or of excluding, based on the knowledge at the time of the analysis, the possibility of a rare genetic disorder (Goh and Choi, 2012). Obtaining a genetic diagnosis for a child can help clinicians and families identify and anticipate future health problems (Wilson et al., 2014), while also informing the health and reproductive choices of family members (Wright et al., 2018).

In pediatric oncology in particular, WGS can inform treatment choices via the characterization of cancers and through the identification of markers relevant for drug metabolism, i.e., pharmacogenomics (Hawcutt et al., 2013). In this context, using WGS to its fullest potential involves comparing the tumor genome to the germline in order to identify cancer-specific genetic variants, implicating both somatic and germline genomic data (Bombard et al., 2013). Thus, WGS technologies help to identify novel genetic alterations contributing to oncogenesis, cancer progression and metastasis, and assist in studying tumor complexity, heterogeneity, and evolution (Shyr and Liu, 2013). The use of genomic sequencing allows for the identification of more effective personalized targeted therapies that lead to increased cure rates and decreased treatment-related morbidity and mortality for the patient affected by relapses or hard-to-treat cancers. Despite these insights, the available clinical care options nevertheless remain insufficient in the case of some pediatric cancers, especially those at advanced stages (Khan et al., 2018).

To overcome these challenges as best as possible, children with cancer may be enrolled in research study such as Terry Fox PROFYLE 2 (PRecision Oncology for Young people 2). PROFYLE 2 targets young patients with difficult-to-treat cancers by sequencing their tumors and, upon recommendation by a molecular tumor board, enrolling those patients in relevant clinical trials. Similar efforts are also underway in other countries (Chakradhar, 2018). In Canada, 17 pediatric oncology centers, in conjunction with the Children's Oncology Group (COG), conduct clinical research studies with the aim to cure and prevent childhood and adolescent cancer through scientific discovery and collaborative research. Some of these studies sequence the child's tumor with the hopes of identifying or testing targeted therapies.

In summary, an increasing number of children with rare diseases and cancer in Canada and around the world are undergoing WGS in clinical or translational research contexts. It is therefore timely to consider the legal framework governing parental requests for access to their child's raw genomic data, and the ramifications of such access for the child's health, privacy, and overall well-being.

## Methodology

The principal method of research employed was doctrinal (Hutchinson, 2018). The two principal laws governing information held by either healthcare institutions^4^ or public bodies^5^ in Ontario were consulted. The statutes were reviewed comprehensively, with a focus on those provisions applicable to parental access to information about their child. Where relevant provisions were found, a search was conducted for related case law from the Information and Privacy Commissioner of Ontario (IPC) or Ontario Superior Courts using WestlawNext Canada and CanLII, two legal databases. IPC and court cases that were responsive to the search were read and analyzed to grasp both the meaning and application of the provisions. We also reviewed the laws that govern personal information held by public- and private-sector organizations of other populous Canadian provinces (Quebec, Alberta, and British Columbia) as well as the European Union. With regards to the former group, a case law search using the legal databases WestlawNext Canada, CanLII, and SOQUIJ was also conducted. Our goal was not to conduct a comprehensive comparative analysis, but to at least hint at the range of potential legal divergence one may expect if our research questions were posed in other jurisdictions.

Raw WGS data includes any one of the common underlying files that are generated in the sequencing process (For a more nuanced introduction to the concept of rawness with regard to genomic data, see Schickhardt et al., 2020). Specifically, this refers to either BCL (base call) files, FASTQ files, SAM (sequence alignment map) files, BAM (binary alignment map) files or VCF (variant call format) files (Evans, 2017). This definition is meant to capture sequence data that has not been subject to any interpretation beyond the data's bare representation, without prejudice to the idea that a representation *per se* implicates an interpretive process (Gitelman and Jackson, 2013). Data having undergone processes such as annotation and interpretation are excluded from this definition (Abril and Castellano, 2019). Return of results and incidental findings are furthermore excluded from this definition of raw WGS data as both such types of information are only generated through the interpretation of sequence data. Nevertheless, as the objects of professional ethical obligations, we also examined the professional guidelines of the American College of Medical Genetics, the Canadian College of Medical Genetics, and the European Society of Human Genetics to understand the relationship of access rights to professional obligations.

## Results

**Figure 1:**
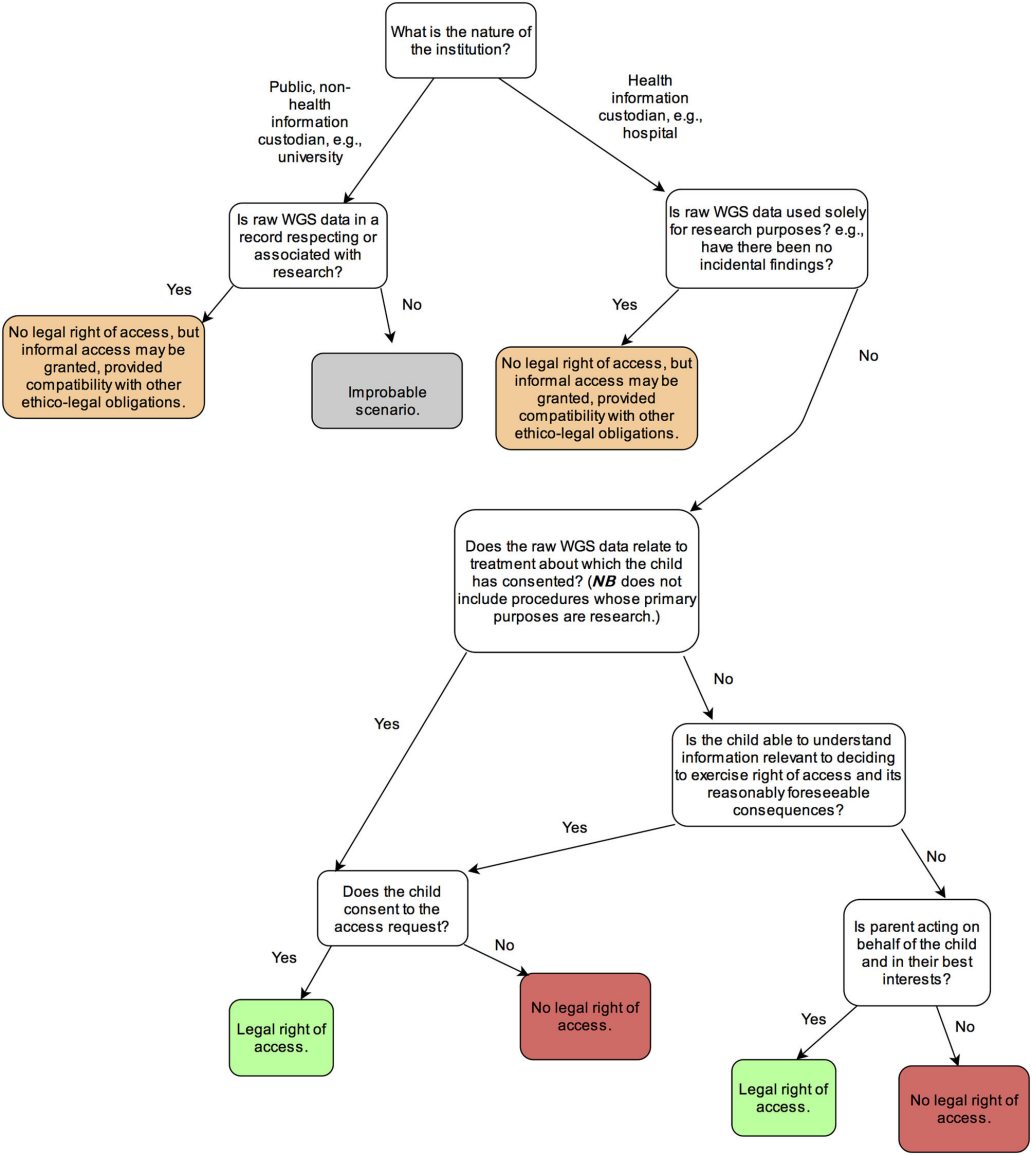
Decisional tree for determining whether parents have a legal right of access to their child's personal information in Ontario.

Our research revealed the following responses to our four legal research questions. First, the right to access health information in Ontario applies generally in healthcare institutions, to both clinical and research data unless the data is held “solely” for research purposes (see Table 1). For all intents and purposes, there is no legal right to access health information associated with research projects at academic institutions. Other provinces and countries may provide broader access rights in research contexts. Regardless, research projects may consider providing access as a matter of policy and ethics while recognizing the limits of such data. Second, access rights in Canada extend to raw WGS data. A patient's legal right of access incorporates raw data, provided their clinician would also hypothetically have access to this data. Third, parents have authority to exercise their child's right of access to health information, as long as they exercise that right *on behalf of* their child. There may be grounds for a health information custodian to refuse parental access requests that are manifestly made to serve the interests of the parent or another party and are not in the best interests of the child. The best interests, however, may not always be an effective ground for constraining parental access requests. Parents are generally given great deference in deciding the best interests of their child (at least under privacy law), as the actions of parents are largely perceived as aligning with their child's best interests. Parental access requests do not necessarily provide sufficient information for health information custodians to assess if access will serve or undermine the child's interests. Finally, children–not their parents–have authority to request access in healthcare institutions if they are over 16 (unless it is demonstrated that the adolescent lacks the capacity to consent), or, even if under 16, if they are sufficiently mature to understand and appreciate the consequences of data access (the mature-minor exception). Where parents request access to information from adolescents, health information custodians may be required to determine if the child is capable of consenting to access before allowing parents to do so. See Figure 1 for a flowchart with the summary of findings.

**Table 1:** Sources and scope of informational access rights in Ontario.

**Name of law and/or regulation**	**Scope/applicability of law and/or regulation**	**Contexts in which a parental access right exists (clinical, research, or both)**	**Applicability of access right to raw genomic sequence data**	**Doctrines that may reduce the scope of parental access right**	**Recognition of “mature minor” doctrine for informational rights**
*Personal Health Information Protection Act*, 2004, SO 2004, c 3, Sch A (“health privacy law”)Complemented by *Ontario Regulation* 329/04.	Private- and public-sector organizations designed as “health information custodians” **Use case:** hospital-based researchers and healthcare professionals acting in a clinical-diagnostic capacity	Clinical: yesResearch: yes, but with narrow exceptions	Yes	Best interests of the child (BIC)	Yes
*Freedom of Information and Protection of Privacy Act*, RSO 1990, c F.31 (“FOI law”)	Public-sector organizations **Use case:** “pure” academic research at a public institution	Clinical: yesResearch: yes, but with broad exceptions	Likely	Best interests of the child (BIC)	No

### Parental Rights of Access to Information About Their Children

The question regarding whether parents have a legal right of access to their child's raw WGS data must be framed through the prism of the general law concerning individuals' abilities to access information about themselves held by others. As a surrogate for the child's interests, parents enjoy a general ability to exercise legal rights *on behalf of* their children, including informational rights^6^^,^^7^. This parental authority is, however, not unfettered, a point which will be developed later through examining how considerations for a child's welfare feature in legal analysis regarding informational rights. What is more, whether personal information is generated for a clinical or research purpose has relevance for the availability of a right to access information (*FIPPA*, s65(8.1); General, O Reg 329/04 (*PHIPA*), s24). To best elucidate the different contours of parental access rights, our legal analysis primarily concerns itself with three different contexts: hospital-based researchers, healthcare professionals acting in a clinical-diagnostic capacity, and “pure” academic researchers at a public institution.

For the three envisaged use cases, there are two relevant laws regarding a parent's potential ability to receive their child's raw WGS data in Ontario: the *Personal Health Information Protection Act* (herein “health privacy law”) and the *Freedom of Information and Protection of Privacy Act* (herein “FOI law”). While there is also the federal *Personal Information Protection and Electronic Documents Act*, SC 2000, c 5 (*PIPEDA*), it applies to only private-sector organizations engaged in commercial activities and who are not also health information custodians under the health privacy law (Office of the Privacy Commissioner of Canada, 2015). Given the wide scope of health information custodians under the health privacy law as well as the public-dominated research and clinical landscape, the situations in which *PIPEDA* applies are limited.

The health privacy law applies only to personal health information (PHI) held by particular custodians of personal health information, which may be either public or private entities. The FOI law, on the other hand, is concerned with information held by public-sector organizations generally. Accordingly, hospital-based researchers and clinicians will generally be subject to the health privacy law whereas academic researchers at universities will be subject to the FOI law.

### Research Exemptions: Tempering Individual Access

Under certain circumstances, both the health privacy law and FOI law exempt information custodians from their obligations to provide an individual with access to their personal information. The health privacy law applies to healthcare institutions and so covers situations where healthcare professionals are acting in a clinical-diagnostic capacity, as well as to hospital-based research. The FOI law applies to public-sector organizations, and so covers academic institutions (see Table 1). Under the health privacy law, access rights apply to health information by hospitals and supporting clinical laboratories. While genetics laboratories are considered health information custodians, only those laboratories where tests are “performed to obtain information for diagnosis, prophylaxis or treatment” are within the ambit of the health privacy law^8^ [*PHIPA*, s3(1)(4)(iv)]. Both laws include exemptions to the access right for research (see Table 2), defined as “a systematic investigation designed to develop or establish principles, facts or generalizable knowledge or any combination of them, and includes the development, testing and evaluation of research”^9^(*PHIPA*, s2). Note, however, that these research exemptions do not prevent researchers from voluntarily providing access to data, provided that such access is compatible with other legal and ethical norms.

**Table 2:** Comparison of research exemptions in Ontario's health privacy and FOI laws.

	**Health Privacy Law**	**FOI Law**
Wording and Scope of Research Exemption	“Personal health information that a researcher uses solely for the purposes of research” is not subject to individual access rights. “Solely” for purposes of research suggests a stringent standard for information to fall within the exemption.	Information contained in records “respecting or associated with research” is not subject to the law. This includes clinical trial data conducted by a person employed by or associated with a hospital.“Respecting or associated with” research requires a substantial connection between the content of the record at issue and specific research being conducted.
Effect of Research Exemption	Exempts research data from the access provisions of the law. The other security safeguards with respect to personal health information continue to apply, however.	Exempts research data from the law in its entirety. No rights and obligations with respect to access, security safeguards, etc.
Situations Where Exemption Lacks Clarity	Individual undergoing whole genome sequencing as part of a research project and sequence data then informs clinical care of the individual, e.g., in cases of incidental or secondary findings.	When data at issue does not implicate academic freedom, e.g., individuals undergoing whole genome sequencing as part of a research project and seek access to this data.

Under the FOI law, records “respecting or associated with” research are exempted from the entirety of the law^10^^,^^11^. This broad exemption is meant to protect the academic endeavor from freedom of information requests, but also exempts individual access rights. Arguably, the research exemption's justification loses its persuasiveness where only an *individual*'s health or WGS information is requested because there is no risk of swiping research results or using research data for other improper purposes (Ries, 2010). Under the health privacy law, the research exemption is narrower—health information used “*solely* for the purposes of research” [our emphasis] is excluded from the access provisions [General, O Reg 329/04 (*PHIPA*), s24(1)]. This suggests that research information also used for clinical-care purposes is subject to the individual's access right [*PHIPA*, s1(b)]. Hospital-based genomics research, especially in pediatric contexts, often has a translational component, where WGS may also be used to assist clinical decision-making (Knoppers et al., 2016; Graaf et al., 2018). Hospital-based research projects that report incidental or secondary findings are likely to be subject to access rights, as the data would no longer be used solely for research purposes. On the other hand, the return of such findings in university-based research would not likely trigger access rights.

It is important to note that the existence and scope of research exemptions vary across provinces and internationally. In Quebec, neither the FOI law nor the law governing public medical records contain a research exemption for access rights^12^^,^^13^. Individuals in Quebec thus appear to enjoy a general right of access to research information about themselves. In British Columbia, one FOI law governs all public entities, including hospitals, but its research exemption only applies to post-secondary educational institutions^14^. Access rights therefore appear to apply to all hospital-based researchers. For universities, Alberta's FOI law follows the same position as British Columbia's FOI law^15^. Alberta's health privacy law is restricted to information related to diagnosis, treatment or care, and so does not have information generated during research as a general concern^16^. Although, as is the case with Ontario's access laws, the robustness the clinical-research distinction may be questioned. Under the European *General Data Protection Regulation* (*GDPR*), access rights apply generally, but Member States are permitted to limit access rights in the research context “in so far as such rights are likely to render impossible or seriously impair the achievement of the specific purposes, and such derogations are necessary for the fulfillment of those purposes” [European Union, 2016, Arts 9(2)(j) and 89]. Such is the case of Germany's *GDPR* implementation; access rights apply to research data unless the access rights are likely to render impossible or seriously impair the achievement of the research (Guerrini et al., 2019; Schickhardt et al., 2020).

### Identifiability and the Characterization of Raw Whole Genome Sequence Data

Assuming no barriers to access rights by way of research exemptions, a legal right to access a child's raw WGS data further requires that the data be considered personally identifiable. For both the health privacy and FOI laws, regardless of clinical or research context, the standard of identifiability is the same: identifying information is information that either directly identifies an individual or information for which it is reasonably foreseeable that it could be used either alone or through combining information to identify an individual [*FIPPA*, s2(1); *PHIPA*, s4(1)]. In clinical care and in many genomic research contexts, genomic and health-related data are either nominally identifiable or coded (a link is maintained between the individual's name and their genomic data), thus maintaining its personal identifiability and consequently allowing for the data to be subject to an access right (Thorogood et al., 2018).

Both the health privacy and FOI laws in Ontario have been found to furnish individuals a legal right of access to raw data, with the IPC having rejected a distinction between raw data and information^17^^,^^18^. Central to this position under the health privacy law is that distinguishing between raw data and information would bring raw data outside of the data protection regime entirely. For genomic sequencing, the distinction between raw data and information would mean that BCL, FASTQ, BAM, and other such files are not subject to the security safeguards and other associated obligations created by law and that only a final lab report or other file resulting from a process of analysis or interpretation would be.

The information governed by the health privacy and FOI laws is nevertheless not coextensive with information to which an individual has a right of access. For example, if a record contains the information of other individuals or information subject to other access exemptions {e.g., quality of care information [*PHIPA*, s51(1)(a)]}, an individual only has access to information that can be reasonably severed (separated) from information to which an individual does not have a right of access [*FIPPA*, s10(2); *PHIPA*, s51(2)].

As regards the health privacy law, information subject to an access right has been found to include raw data from diagnostic equipment from which information in an individual's health record had been derived^19^. A guiding principle in determining which information an individual has access to under the health privacy law is informational reciprocity in the clinician-patient relationship^20^. Hence, a patient has a legal right of access to any data, including raw data, to which their clinician would also reasonably have access. For example, an individual would not have a right of access to raw data used in machine processing and that a clinician cannot reasonably use^21^. Furthermore, an individual's access right extends to data that may be extracted via custom queries using currently available software and formats, but not if accessing the information would require the development of novel methods^22^.

In Alberta, British Columbia, and Quebec, an individual's right of access generally extends to raw data about themself^23^^,^^24^^,^^25^. Only in Quebec, however, has it been found that individuals also have a legal right to access raw clinical data about themselves, even where an individual has already received a report based on the interpretation or analysis of this data^26^. The underlying logic behind this position is that the governing law does not distinguish between raw data and other types of information. Consequently, raw data forming part of an individual's health record can be the object of an access request. Information and privacy commissioner decisions in Alberta and British Columbia have not dealt with raw health-related data other than in the context of psychological tests. Given that no distinction exists between raw data and information as a matter of law, it appears strongly probable that, when faced with an individual access request to raw health-related data, an access right to raw data will be recognized. Indeed, in the European context, it has been argued that under the *GDPR*, individuals have a general right to raw data about themselves (Schickhardt et al., 2020).

As with the case in Ontario, an individual's right of access to raw data is not unfettered. For example, Quebec's FOI law does not give individuals a right of access to documents that require “computation or comparison of information”^27^. That is, an information custodian does not need to create a document or file of assembled information solely for the purposes of providing access^28^. Extracting data from an information system does not, however, constitute the creation of a new document^29^. In contrast, laws in British Columbia and Alberta require that the information custodian create a record for an individual exercising an access right^30^^,^^31^^,^^32^. However, and much as is the position under Ontario's health privacy law, individuals in Alberta, British Columbia, and Quebec do not have a right of access to data to which the information custodian does not have access through existing software and/or normal technical expertise^33^^,^^34^^,^^35^^,^^36^^,^^37^. Moreover, it is a commonality that the information must be reasonably severed if contained in a record not dedicated primarily to the individual's personal information^38^^,^^39^^,^^40^^,^^41^. Applied to raw WGS data, it then seems that an individual will have an access right to their raw WGS data, pending no other potential exclusions, as explored in the next sections.

### Best Interests of the Child: A Limit on Parental Access?

The best interests of the child (BIC) is a legal standard whose most important source is the *CRC*. The BIC is at the forefront of legal and ethical considerations in making decisions concerning a child (Sénécal et al., 2015b). The *CRC* itself deems BIC the “basic concern” of parents (United Nations General Assembly, 2007, 18). In Canada, “the values and principles of the Convention recognize the importance of being attentive to the rights and best interests of children when decisions are made that relate to and affect their future”^42^.

The multifactorial, context-specific nature of the BIC has been criticized for its failure to produce clear, bright-line rules (Parker, 1994). It is, however, largely by virtue of the BIC's context-specific nature that gives it its potential to be applied using localized meanings and understandings in a way that serves its overarching purpose—the treatment of the child in a way that promotes their welfare while also being responsive to the child's age and capacities (Parker, 1994; Lansdown, 2005).

The BIC standard is relevant for the exercise of informational rights, and in particular the right of access. BIC has been applied in the context of access requests under both the health privacy and FOI laws. The approach of applying BIC under both laws is the same and can act as a limit on parents' ability to access information about their children^43^^,^^44^. For example, the Ontario IPC has found that a father making an information access request, despite having done so in good faith, was nevertheless not acting *on behalf of* the child, but rather for his own collateral purpose and so access to the information at issue was not granted^45^. The adjudicator further found, “based on the sensitive nature of the materials contained in the records, that the release of the son's personal information would not serve the best interests of the child.”^46^ The decision's reasoning that the exercise of rights *on behalf of* a child requires a connection to that child's best interests finds further support in Ontario's *Children's Law Reform Act*, which states that incidents of custody of the child, such as exercising an access to information right, are to be determined with reference to the BIC^47^. In this way, the intersection of BIC and informational rights ensures that the parent is in fact acting on behalf of the child in a way that coheres with that child's best interests.

Beyond Ontario, the BIC remains a primordial consideration in all decisions concerning a child. As in Ontario, however, each province had limited case law concerning the intersection of the BIC standard and informational rights. In Quebec, the Civil Code requires that all decisions concerning a child take into account that child's interests and rights, including the right of the child to be involved in the decision-making process in a way that is compatible with their maturity^48^. British Columbia similarly has found that acting on behalf of a child is synonymous with acting in the best interests of the child, even where informational rights are concerned^49^. The BIC is furthermore relevant in Alberta, where the disclosure of confidential information may be justified if it is in the child's best interests^50^. Similarly, the BIC and the child's right to be involved in decisions affecting themself are fundamental rights in the European Union (European Union, 2012, Art 24). The intersection of child's rights and informational rights has nevertheless garnered criticism on the basis that the child's evolving capacities are not adequately taken into consideration (Buitelaar, 2018).

### Mature Minors in Access Contexts

Children are both bearers of rights and in need of protection owing to their vulnerability. As they age and mature, children present distinctive rights and needs, in the informational context and elsewhere. Central for our purposes is the *CRC*'s “participatory/emancipatory concept,” whereby rights are transferred from the parent to the child in recognition of the child's developing maturity (Lansdown, 2005). Concern for the child's developing autonomy finds its principal expression in the involvement of the child in decision-making processes, such as the informed consent process (United Nations General Assembly, 1989, Art 12). The informed consent process must mediate between concerns for a child's developing autonomy, self-awareness, values, ability to understand, and the overarching concern for a child's best interests (Coughlin, 2018). Elucidating this mediation process, the Supreme Court of Canada has stated that the BIC “must be interpreted in a way that reflects and addresses an adolescent's evolving capacities for autonomous decision-making.”^51^ For children who possess a high level of maturity, the concerns for the child's welfare (concretized in the BIC standard) on one hand, and their autonomy on the other, “will collapse altogether and the child's wishes will become the controlling factor.”^52^

Assuming that an access right exists in relation to the information, an information custodian must determine who is capable of exercising the right. In what follows, we examine the rights of access of children and of parents under both the FOI and health privacy laws (see Figure 1 for summary). We will show that the two laws share a common point of departure—parents may exercise access rights where the child is under 16 years of age. But there is a lack of clarity regarding cases where the sampling and sequencing procedure serves both clinical and research purposes or the procedure is undergone solely for research purposes.

For the purposes of our analysis, we assume that the parent requesting access is a custodial parent and that the child to whom the WGS data relates follows a unidirectional, progressive trajectory with regard to their capacity for autonomous decision-making, i.e., that capacity is not present at one time but then is lost at a later time. We use the term “mature minor” in the narrow sense to refer to the mature minor legal doctrine, as well as in the broad sense to refer to minors who either have capacity to consent to treatment or who have informational capacity.

Both the FOI and health privacy laws share a common starting point: where a child is under 16 years of age, the parent or other LAR may exercise the right of access on behalf of the child (*FIPPA*, s66; *PHIPA*, s23). Contrary to the health privacy law, the FOI law does not incorporate the mature minor doctrine into its access provisions. The FOI law's bright-line approach, whereby parents exercise informational rights on behalf of a child under 16 years of age without regard to the circumstances, may be understood as fusing the interests of parent and child [*FIPPA*, s66(c)]. The only potential for a separation of the interests of the parent and child is through reference to the BIC standard (*infra*).

The health privacy law contains two key exceptions to the general rule that a parent or LAR may exercise the right of access on behalf of a child under 16 years of age. The first is that parents and other LARs do not have a right of access where the information relates to treatment or care to which the child has consented on their own [*PHIPA*, s23(2)(i)]. The health privacy law works in concert with the law governing capacity to consent to clinical treatment, ensuring that informational rights traces authority with regard to clinical decision-making (*HCCA*). The second exception to the general rule that parents or other LARs have a right to access information about a child under 16 years of age concerns minors who are informationally capable. We explore each in turn.

In Ontario, all individuals, including children, are presumed to be capable of consenting, unless the individual is unable to understand information relevant to the treatment^53^. Capacity to consent to treatment revolves around the notion that treatment, “means anything that is done for a therapeutic, preventive, palliative, diagnostic, cosmetic or other health-related purpose,” (*HCCA*, s2). Capacity is determined on a treatment-by-treatment basis with regard to the capacity of the patient to understand the information relevant to that decision and to appreciate the associated reasonably foreseeable consequences (*HCCA*, s4). Consequently, a minor may be competent to consent to a low-risk procedure such as the removal of a mole, while for higher risk procedures, such as novel chemotherapies, that same minor would not be competent to consent. Where a mature minor has consented to a sampling and sequencing procedure for a clinical-diagnostic purpose, a parent does not have a right of access to any of the sequence data, raw or otherwise, unless the minor consents to releasing the information to the parent.

For a procedure whose “primary purpose” is research, however, the general rules regarding parental access under the health privacy law apply. Consequently, if the child is under 16 years of age and the primary purpose of the sequencing and sampling was research, then the parent will have *prima facie* a right of access to that information. The breadth of scenarios covered by the primary purpose criterion is broad. Where a child undergoes sampling and sequencing as part of participation in a research study in the hope that the data generated will be relevant for treatment, the primary purpose appears to remain research as the research study is the reason for which the data is generated. Any potential clinical application is merely secondary. This is significant as the primary purpose criterion implicates both pure research and research-clinical scenarios. Recall that, as regards the former, the return of incidental findings should hypothetically trigger an access right and that the research exemption would not apply to the latter because such information would no longer be used exclusively for research purposes.

The effect of the foregoing is that where adolescent children undergo a sampling and sequencing procedure, a parent will *prima facie* have a right to data generated in either the pure research or research-clinical contexts, but not to data generated in relation to the clinical-diagnostic context. Informational rights do not trace decisions by a minor regarding research participation because there is no legal mature minor doctrine for research participation. As an ethical process, assent does not directly affect legal rights with regard to information related to research.

The second exception to the general rule that parents or other LARs have a right to access information about a child under 16 years of age concerns minors who are informationally capable. Where a minor child is able to “understand the information that is relevant to deciding whether to consent” and to “appreciate the reasonably foreseeable consequences” of a decision regarding their information, they are recognized as having capacity for the purposes of the health privacy law (herein “informational capacity”) [*PHIPA*, s21(1)]. Where the decision of a parent or other substitute decision-maker differs from a capable child as regards that decision, the child's decision prevails over the conflicting decision of the parent^54^ [*PHIPA*, s23(3)].

Informational capacity gives the minor a voice, even where they have not consented to the procedure to which the information relates. However, it introduces complexities for information custodians. Examining whether the child consented to the procedure at issue is only a first step. Even if they did not, they may still possess informational capacity such that the access right must be exercised by the child themself. The relevant point in time for undertaking the analysis is at the time of the access request. Consequently, an adolescent may be likely to possess informational capacity for information that relates to a procedure they underwent in their tender years.

One may still question the significance of informational capacity in practice. Consider that when a minor's decision regarding treatment or care is at issue, the clinician has directly interacted with the minor-patient and so is in an appropriate position to judge that minor's capacity to make a choice for treatment. Yet in the informational context, an access request will likely be handled by an administrator without personal knowledge of the child to whom the information relates. There is no explicit obligation for an information custodian to determine whether a child is informationally capable upon receipt of an access request from a parent (Perun et al., 2005). It appears likely that, unless an information custodian knows that a minor disagrees with a parent's access request, access is likely to be granted. The general duty of information custodians to act in “in good faith and reasonably in the circumstances” may, however, give rise to an obligation for custodians to take into consideration the minor's decision-making capacity at the time of the access request [*PHIPA*, s71(1)].

The circumstances under which a parent has access to their child's information varies widely by province in Canada. For consent to clinical care, Quebec follows an age-based criterion that presumes any individual above 14 years of age may consent to treatment required for their health unless there is reason to believe the individual does not have sufficient decision-making capacity^55^. Similar to Ontario, informational rights map onto this age of consent: minors over the age of 14 have access rights under the statute that governs individuals' medical files^56^. Where a parent requests access to information in a medical record that relates to a child who is 14 or older, the custodian must first consult the child and the child's decision regarding whether or not to provide access to the parent is binding^57^.

Other provinces follow the “mature minor” doctrine, initially developed in England and Wales, whereby a minor who is capable of understanding the proposed course of clinical action and is capable of expressing their own wishes regarding this course of action may consent to care, provided that it is in their best interests and notwithstanding their general lack of legal capacity due to their age (e.g., British Columbia and Alberta)^58^^,^^59^^,^^60^^,^^61^ (Dalpé et al., 2019). In British Columbia, a parent may only exercise a child's access right where the child is incapable of exercising it themselves^62^. In practice, informational competence tends to be recognized at the age of 12 and so parents require their child's authorization to access their health files^63^ (see, e.g., Health Information Management, 2020). Likewise, Alberta's health privacy law is also consistent with the mature minor doctrine regarding consent to clinical care. Under Alberta's health privacy law, a parent making an access request for information about their child under the age of 18 bears the onus of proving that their child “does not understand the nature of the right…and the consequences of exercising the right” at the time that the request is made^64^^,^^65^. Notably, “the level of understanding that is required for an individual to understand the nature and consequences of exercising rights or powers under [Alberta's health privacy law] is not a particularly onerous standard,”^66^. The kind of information at issue appears to be irrelevant for the purpose of determining informational capacity. Thus, there is no clear support that there is a higher capacity required for exercising access rights over raw WGS data than other kinds of health information. Alberta's FOI law takes a novel approach in that a parent may exercise the child's rights provided that such exercise does not cause “an unreasonable invasion of the personal privacy of the minor,” which presumably is intended to be a case-by-case determination with the minor's evolving capacities taken into account^67^. The position under the *GDPR* is largely a question for Member State law, as the regulation only makes specific provision for the age at which children may consent in relation to information society services, and not to data processing activities generally (European Union, 2016, Art 8). In view of the wide diversity of approaches and the multiple considerations at play (e.g., age of consent to clinical treatment, to research, to data processing, duties to assess a minors' capacity before allowing parents to exercise their rights, duties to consult minors before releasing data, etc.), health professionals should carefully consider the potential interface of these factors under local law.

## Discussion

Individuals in Ontario have a legal right to access their genomic data used for clinical and translational research. Ethical and legal literature in genomics has mainly focused on the obligations of professionals implicated in the bioinformatics pipeline with respect to test interpretation and the return of incidental or secondary findings, with raw data receiving less attention (Borry et al., 2018). Previous articles in the Canadian context have focused on access requests in the context of academic health research (Ries, 2010), or on individual control over genetic information (Ogbogu et al., 2014). We expand upon these articles by identifying legal access rights to clinical data and by clarifying the scope of research exemptions in Ontario. In healthcare institutions, only data solely used for research is exempted from the individual right of access. In academic institutions, all data associated with research is exempted. We also find case law indicating that access rights should encompass raw WGS data. Exempt research projects can still decide to offer access as a matter of ethics and participant engagement.

Our study is the first to trace the contours of parental access rights where children undergo WGS. We find that parents' authority to request access must be exercised on behalf of the child and in that child's best interests. Health information custodians would have grounds to refuse an access request manifestly not in the child's best interests. Furthermore, we find that health information custodians likely have a duty to ensure parents are not granted access to a mature minor's information, unless the minor consents or the parent demonstrates that the minor lacks capacity to make decisions about the disclosure of their health information. The position concerning access rights is most clear in the case of sequencing for exclusively clinical-diagnostic purposes across jurisdictions, albeit with importance nuances among them. Parental access to research data typically is not possible, due to the research exemptions. If it is possible, however, the provinces also differ greatly in their approaches.

Despite the clear evidence of BIC's relevance for the exercise of informational rights, the cases in which BIC has been applied in the context of parental access requests are limited. Indeed, this was true across all Canadian provinces under study. The small number of cases suggests either that information custodians do not generally deny parental access requests or that such denials are not appealed to the provincial IPC (Cases only appear in front of the provincial IPC in circumstances where an individual is challenging a decision made by an information custodian). It is moreover difficult to envisage the circumstances in which an information custodian would be able to easily distinguish between the BIC and any ulterior motives on the part of a parent. For example, one of the few cases where BIC was an express consideration was when a parent had requested information from a police report on behalf of their children, but the children were fearful of them and did not desire contact with the parent^68^. It thus seems that the BIC standard exists in principle in relation to information access requests, but the circumstances in which information custodians may meaningfully invoke it are limited. Importantly, we note that the vast majority of parents are likely making decisions that are in keeping with their child's best interests. As such, information custodians should not be quick to second-guess parental motives in most circumstances. To this end, see section Conclusion and Points to Consider for recommendations in.

### Leveraging Professional Expertise With Access Rights

While law provides an important framework in this area, ensuring parental access supports the welfare, privacy, and developing autonomy of children will primarily depend on the ethical behavior of both professionals and parents. One important challenge for information custodians and professionals is the difficulty of distinguishing beneficial parental access requests from improper ones. Likewise, it may be difficult to craft legislation or professional guidelines that effectively make this distinction. Too much intervention risks depriving children of their right to receive parental guidance in keeping with their age and capacities.

The existence of legal access rights, rather than trumping professional obligations, invites us to reconsider how the child's best interests can be furthered. Professional expertise should be leveraged to further the child's best interests, which should include the involvement of the most important individuals in a child's life—their parents. Professionals should thus engage with parents and help them decide if access is the right decision for their child, and how to responsibly handle the data once accessed. The potential for parental access may also encourage professionals to more carefully consider whether or not to sequence children in the first place. Ultimately, much of the responsibility to act on behalf of, and thus safeguard, the child's interests will rest with parents. Careful management of a child's personal information is an increasingly important parental responsibility–this responsibility also extends to genomic data.

A fruitful starting point in ensuring that the exercise of access rights is in keeping with the child's best interests are professional guidelines developed to address the return of incidental or secondary genomic findings in children. These guidelines highlight the ethical challenges with respect to handling the genomic data of children, particularly where it reveals health risks that may only materialize after the child has reached maturity. On the one hand, returning predictive information to children and their parents may inform childhood or adulthood actions that could improve the child's future health (Johnson et al., 2017). The return of information may also better inform the health choices of family members, which can improve the overall well-being of the child (Hardart and Chung, 2014). On the other hand, returning predictive information may threaten the child's future autonomy and ethical right not to know (Feinberg, 1980). Return may also lead to psychological harms (e.g., anxiety, low self-esteem), harms to family relationships, and potential discrimination (McGuire et al., 2020). Flowing from these competing concerns, professional guidelines have made different recommendations about the reporting of adult-onset genomic findings in pediatrics.

Clinicians using WGS tests may look to their professional associations for guidance on how to deal with requests to provide parents access to their child's raw WGS data. The American College of Medical Genetics (ACMG), the Canadian College of Medical Genetics (CCMG), and the European Society of Human Genetics (ESHG) have not published any policies on responding to access requests to raw data generally (“pulling” data). Nevertheless, each organization does have a position on the return of incidental or secondary findings, i.e., “pushing” data (Green et al., 2013; van El et al., 2013; Boycott et al., 2015).

Secondary findings describe pathogenic variants that are identified in the genome of a patient unrelated to the primary purpose of the testing (Knoppers et al., 2015). Secondary findings and raw data are undoubtedly distinct from one another: the former are curated (and, thus, the product of an interpretive process) and the latter are merely the subject-matter of that interpretive process. However, both represent different forms of genomic information that can be returned to individuals if requested. With this common characteristic considered, and in the absence of any guidance on the return of raw data, it is worth briefly exploring positions on the return of secondary findings.

The ACMG has the most permissive policy on returning secondary findings, recommending that a predetermined list of variants associated with medically actionable disorders be returned to patients, provided consent is obtained, in addition to primary test results (Green et al., 2013). Importantly, while the majority of these conditions are adult-onset, the ACMG also recommends returning these variants when found in children as the results may have immediate implications for family members and for the child when they are older. The ACMG also highlights the importance of parental decision-making when it comes to genetic testing. Despite its nuances, this approach not been without detractors (Garrett et al., 2019).

The ESHG and CCMG take a more cautious and classical approach by suggesting the creation of a bioinformatics pipeline that minimizes the identification of secondary findings (van El et al., 2013; Boycott et al., 2015). The CCMG nevertheless recognizes that labs may want to search for secondary findings and provide guidance on what results to return. They suggest that labs searching for secondary findings ought to return results for highly penetrant conditions that are medically actionable in childhood. Variants associated with adult-onset medically actionable conditions should only be returned upon request, when the data has the potential to prevent serious harm to the health of a parent or family member. The ESHG highlights concerns over respecting the emerging autonomy of children, while the CCMG suggests that there may be psychosocial harms associated with returning secondary findings as a rationale for their cautious approach.

While providing secondary findings and returning raw data both involve returning genomic information that may have nothing to do with the primary indication for testing, the scale of data being returned is vastly different. For example, the ACMG suggests screening for pathogenic variants in only 59 genes (Kalia et al., 2017). In contrast, raw genomic data contains information on all genes and intervening sequence in the genome. Returning raw data could be considered analogous to returning all variants, depending on what is done with the data. Raw data could be analyzed to identify variants associated with adult-onset non-medically actionable diseases, variants of unknown significance, and the carrier status of the child. To our knowledge, no professional guideline or policy has even contemplated returning this type of information to parents.

Despite the silence of professional norms regarding the return of raw sequence data, many laboratories performing clinical WGS permit raw data release. A recent study examined the content of publicly available consent forms to determine whether they complied with recommendations made by the ACMG and the Presidential Commission for the Study of Bioethical Issues (Fowler et al., 2018). Germane to this discussion was the recommendation made by the Presidential Commission that patients be informed of what data and information may be returned (Presidential Commission for the Study of Bioethical Issues, 2012). Of the 18 consent forms analyzed, 44% provided for return of the raw data to the clinician, with commercial laboratories being more likely to permit raw data release compared to academic labs (Fowler et al., 2018). This study suggests that a large minority of patients are made aware that raw data release is possible and that clinicians are the gatekeepers for this information. Regardless, patients generally have legal rights to access health information held by laboratories in Ontario either directly or indirectly through their clinician^69^.

In this vein, laboratory data retention practices are noteworthy. Despite health information retention laws, and professional recommendations for retention of some data files by clinical genetics laboratories, both policy and practice remain unclear and variable. For example, the CCMG recommends that clinical genetics laboratories retain the VCF file for a minimum of 2 years and possibly even longer for the testing of minors or for inherited disorders with familial implications (Hume et al., 2019). Surprisingly, the CCMG's recommendation of retaining a VCF file for at least 2 years is markedly shorter than the periods established by other legal and ethical norms for retention of health information, e.g., 10 years in the case of health information and 5–10 years for diagnostic imaging records^70^^,^^71^. One possible reason for this discrepancy could be that clinical genetics laboratories do not typically have direct contact with patients and the VCF file represents an intermediate step between the act of sequencing and the information relayed to a patient by their clinician. The existence or accessibility of the file over time clearly has implications for parental access.

Our analysis of legal rights of parental access is connected to another debate regarding parents' ability to have their child tested through direct to consumer (DTC) genetic testing services. Usually, children are only sequenced in health care where there is an important clinical indication, and in research where there is a need to improve our understanding of serious childhood conditions. With DTC, parents can seek genetic testing of healthy children or children with non-serious health conditions. Some of the health information they may receive, such as information about adult-onset conditions, raise the ethical issues highlighted above between access to health information for children and families, and closing of the child's future choices not to know their health status. Furthermore, parents can generally access their child's genetic data in the DTC context because *PIPEDA* sees the parent effectively exercising the child's legal informational rights on behalf of the child and does not expressly consider the rights of children with regard to access. Parents may then share the child's data with third party interpretation services, clinicians, researchers, and even open-access recreational genomics sites. While this may offer interesting health, research, and recreational opportunities for both parents and children, there is also the potential for important privacy risks and discrimination.

With the increase in sequencing in the research and clinical contexts, coupled with the advent of DTC genetic testing services, parents have greater freedom to test their children for various health risks and to direct the sharing of their children's data. A recent study counted as many as 35 raw genomic data interpretation services available to consumers online (Capaci et al., 2020). Parents are already attracting more responsibilities for safeguarding their children's privacy with their social media interactions. Such responsibilities are likely to extend to understanding the health and privacy implications of genetic testing and data sharing for children.

Our study focused on describing the application of current law to parental genomic access requests. Future legal studies could explore if laws should be adapted to be more responsive to the challenges of genomic and children's privacy. Future legal research questions include the following: Are individual access rights an appropriate and effective way to empower patients? For example, it has been highlighted that proactive approaches of providing individuals with access to their data would be better for all parties involved, as formal access requests are clunky and time-consuming (Kwoka, 2017). Should health privacy laws incorporate more explicit protection and consideration for the child's best interests? (Buitelaar, 2018; Savirimuthu, 2019) Is direct regulation of parents regarding their children's genetic data desirable? Feasible? What about greater regulation of third-party interpretation services, especially when it comes to children's genetic data? (Guerrini et al., 2020)

## Conclusion and Points to Consider

Health professionals, researchers, and their organizations must carefully consider the legal and ethical implications of parental access when handling requests or designing personal genomic access policies and processes. They need to be able to determine when parents have a legal right to access their child's health information, the ethical implications of parental access for the child, and their corresponding professional duties to protect the best interests and developing capacity of young patients and research participants.

While our study has focused on the legal rights of access of parents to their child, the avenues of inquiry may be generalized for other jurisdictions. Individuals should identify the controlling legal framework regarding individual access rights, which will most often be contained in either privacy and data protection or freedom of information laws. It is also essential to determine the existence and ambit of any exemptions in the research context. Furthermore, individuals should examine if raw data constitutes personal information under relevant privacy and data protection and/or freedom of information laws. Where parents are requesting the personal information of their children, two additional and interrelated issues are present: the BIC and provisions for “mature minors”/the need to involve a child in appropriate manner based on age and competence. Either of these aspects of children's rights may temper the parental access right.

General recommendations for personal genomic access in healthcare and health research contexts have already been developed in the German context by the Ethical and Legal Aspects of Whole Genome Sequencing project (EURAT) (Winkler et al., 2019). We endorse EURAT's core recommendations of pre- and post-access education. Such an approach sees professional expertise working together with legal rights to further the health, privacy, and general welfare of probands and their families. EURAT recommends that an initial conversation be held with requestors to explain the access process and assess their capacity and motivation. At this stage, general information about the nature and implications of raw genomic data should be provided to help requestors determine if access will serve their purposes. This information may include disclaimers about quality and fitness for medical use, information about the limited meaning of the information, the need for expert interpretation, and the health and privacy risks to the individual and family members that can arise from sharing genomic data. The individual can then be offered an opportunity for sober reflection and reconsideration after this initial conversation. If the individual proceeds with the request, they can be offered general written information about the health and privacy implications of the raw data should be provided, as well as an opportunity for personal consultation, while making it clear this is not individualized genetic counseling. Each of these steps should be carefully documented.

Overall, EURAT also recommends that healthcare organizations and research projects should establish a clear and accessible policy to facilitate handling of requests, describing the scope of the right to access, the process for requesting access, and opportunities to receive information and consultation. Moreover, appropriate quality-control mechanisms for sample and data tracking and identity authentication processes must be in place to ensure the right data is returned to the right person. One final general consideration is that access requests should be directed through the ordering physician, and not the laboratory directly.

While a useful source of guidance, EURAT's recommendations are neither specific to the pediatric context nor to the unique contours of legal access rights in Canada. As such, we propose these additional considerations:

- If possible, professionals in the child's circle of care should speak to parents who are requesting access to raw sequence data to better understand the context of the request. It may turn out that the parents' request may be better satisfied by other avenues, e.g., returning interpreted results. An explanation of the interpretation processes the sequence data have already undergone can assist parents in understanding the nature of their request. For example, if a search has already been conducted for highly penetrant conditions that are clinically actionable in childhood, parents may decide that having the raw sequence data is not needed.
- Pre- and post-access informational materials and consultations should inform parent requestors about the implications of raw data for the child's well-being, privacy, and developing autonomy. They should also inform parental requestors of their ethical responsibilities for handling, using and sharing their child's genome responsibly.
- Information custodians should withhold access if it is manifestly clear to the professional that the parent is not acting on behalf of the child, viz. for an ulterior purpose such as uploading the child's sequence data to an online portal for a reason disconnected from the child's best interests. Nevertheless, we recognize that professionals may rarely have clear evidence about the motivations to justify refusing parental access. Moreover, parents can always lodge an appeal to an information custodian's decision with which they disagree.
- Steps should be taken to determine that only the individual who is legally authorized to exercise the child's access right is permitted to access data (parent, mature-minor, LAR, or no one). This will generally be determined by the age of consent, but also exceptionally by the child's level of maturity in the clinical context. We provide a flow chart to aid with this determination (see Figure 1). In particular, health information custodians should consider an older child's developing maturity: they should seek to determine if the child has the capacity to exercise informational rights alone before granting access to parents, and to ensure the child has been consulted about the request in an age-appropriate manner.
- In pediatric research contexts where there is no legal right to access, a governance decision should be made before recruitment as to whether or not the project will provide access to parents, considering the consequences for research integrity, available resources, and expectations of participants. The specific research context may be important. Parents of sick children with rare diseases, chronic conditions, or cancer may deserve greater deference in managing their child's genetic data in order to drive their care and related research, than parents of healthy children. If providing access may bias research outcomes, then access may require the participant to withdraw from the study. If a research project voluntarily opts to provide access, the considerations above for doing so responsibly are applicable.

## Author Contributions

MB conducted the doctrinal review. MB and AT interpreted the data. MB, AT, MS, and KS drafted the manuscript while critical revision was provided by MZ and BK. All the authors approved the manuscript for publication.

## Conflict of Interest

The authors declare that the research was conducted in the absence of any commercial or financial relationships that could be construed as a potential conflict of interest.

## Footnotes

^1^Genetic Alliance Promise for Engaging Everyone Responsibly|GeneticAlliance.org. Available at: http://www.geneticalliance.org/programs/biotrust/peer (Accessed February 1, 2021).

^2^Department of Health and Social Care (England) Health minister: NHS must lead the world in genomic healthcare. GOV.UK. Available at: https://www.gov.uk/government/news/health-minister-nhs-must-lead-the-world-in-genomic-healthcare (Accessed February 15, 2020).

^3^*McInerney v*. MacDonald, 1992 CanLII 57 (SCC).

^4^*Personal Health Information Protection Act*, 2004, SO 2004, c 3, Schedule A.

^5^*Freedom of Information and Protection of Privacy Act*, RSO 1990, c F.31.

^6^*Freedom of Information and Protection of Privacy Act* (*FIPPA*), RSO 1990, c F.31, s66(c).

^7^*Personal Health Information Protection Act (PHIPA)*, 2004, SO 2004, c 3, Schedule A, s23.

^8^*Laboratory and Specimen Collection Centre Licensing Act*, RSO 1990, c L.1, s5.

^9^*McMaster University (Re)*, 2008 CanLII 36902 (ON IPC).

^10^*Carleton University v. Information and Privacy Commissioner of Ontario and John Doe, requester*, 2018 ONSC 3696, 2018.

^11^*McMaster University (Re)*, 2008 CanLII 36902 (ON IPC).

^12^*Act respecting Access to documents held by public bodies and the Protection of personal information*, CQLR c A-2.1.

^13^*Act respecting health services and social services*, CQLR c S-4.2.

^14^*Freedom of Information and Protection of Privacy Act*, RSBC 1996, c 165, s3(1)(e).

^15^*Freedom of Information and Protection of Privacy Act*, RSA 2000, c F-25, s4(1)(i).

^16^*Health Information Act*, RSA 2000, c H-5, ss1(1)(i) and 1(1)(k).

^17^*Ontario (Natural Resources) (Re)*, 2003 CanLII 53917 (ON IPC).

^18^*St. Michael's Hospital (Re)*, 2017 CanLII 70006 (ON IPC).

^19^*Family Services of Peel (Re)*, 2019 CanLII 75908 (ON IPC).

^20^*St. Michael's Hospital (Re)*, 2017 CanLII 70006 (ON IPC).

^21^*St. Michael's Hospital (Re)*, 2017 CanLII 70006 (ON IPC).

^22^*St. Michael's Hospital (Re)*, 2017 CanLII 70006 (ON IPC).

^23^*Alberta Innovates – Technology Futures (Re)*, 2012 CanLII 70603 (AB OIPC), 2012.

^24^*G.F. c. Centre de réadaptation en déficience intellectuelle et en troubles envahissants du développement du Saguenay-Lac-Saint-Jean*, 2015 QCCAI 160 (CanLII).

^25^*Ministry of Forests, Re*, 2003 CanLII 49186 (BC IPC).

^26^*G.F. c. Centre de réadaptation en déficience intellectuelle et en troubles envahissants du développement du Saguenay-Lac-Saint-Jean*, 2015 QCCAI 160 (CanLII).

^27^*Act respecting Access to documents held by public bodies and the Protection of personal information*, CQLR c A-2.1, s15.

^28^*C.S. c. Société de l'assurance automobile du Québec*, 2017 QCCAI 251 (CanLII).

^29^*Stratégie 360 inc. c. Laval (Ville de)*, 2012 QCCAI 238 (CanLII).

^30^*Freedom of Information and Protection of Privacy Act*, RSA 2000, c F-25, s10.

^31^*Freedom of Information and Protection of Privacy Act*, RSBC 1996, c 165, s6.

^32^*Health Information Act*, RSA 2000, c H-5, s10.

^33^*Act respecting Access to documents held by public bodies and the Protection of personal information*, CQLR c A-2.1, s15.

^34^*Freedom of Information and Protection of Privacy Act*, RSA 2000, c F-25, s10.

^35^*Freedom of Information and Protection of Privacy Act*, RSBC 1996, c 165, s6.

^36^*Health Information Act*, RSA 2000, c H-5, s10.

^37^*Stratégie 360 inc. c. Laval (Ville de)*, 2012 QCCAI 238 (CanLII).

^38^*Act respecting Access to documents held by public bodies and the Protection of personal information*, CQLR c A-2.1, s14.

^39^*Freedom of Information and Protection of Privacy Act*, RSA 2000, c F-25, s6(2).

^40^*Freedom of Information and Protection of Privacy Act*, RSBC 1996, c 165, s4(2).

^41^*Health Information Act*, RSA 2000, c H-5, s7(2).

^42^*Baker v. Canada (Minister of Citizenship and Immigration)*, 1999 CanLII 699 (SCC), para 71.

^43^*Family Services of Peel (Re)*, 2019 CanLII 75908 (ON IPC).

^44^*Ontario (Community Safety and Correctional Services) (Re)*, 2016 CanLII 25549 (ON IPC).

^45^*Ontario (Community and Social Services) (Re)*, 1994 CanLII 6595 (ON IPC).

^46^*Ontario (Community and Social Services) (Re)*, 1994 CanLII 6595 (ON IPC), p 3.

^47^*Children's Law Reform Act*, RSO 1990, c C.12, s19(a).

^48^*Civil Code of Québec*, CQLR c CCQ-1991, 1991, Arts 32–34.

^49^*British Columbia (Children and Family Development) (Re)*, 2018 BCIPC 47 (CanLII).

^50^*High Prairie School Division No. 48 (Re)*, 2012 CanLII 70631 (AB OIPC).

^51^*A.C. v. Manitoba (Director of Child and Family Services)*, 2009 SCC 30 (CanLII), para 88.

^52^*A.C. v. Manitoba (Director of Child and Family Services)*, 2009 SCC 30 (CanLII), para 87.

^53^*Health Care Consent Act* (*HCCA*), 1996, SO 1996, c 2, Sch A.

^54^*Family Services of Peel (Re)*, 2019 CanLII 75908 (ON IPC).

^55^*Civil Code of Québec*, CQLR c CCQ-1991, 1991, Arts 14 and 17.

^56^*Act respecting health services and social services*, CQLR c S-4.2, ss17, 20, 21.

^57^*Gagné c. Hôpital Ste-Justine* [1999] CAI 261.

^58^*Infants Act*, RSBC 1996, c 223, 1996, s17.

^59^*A.C. v. Manitoba (Director of Child and Family Services)*, 2009 SCC 30 (CanLII).

^60^*Gillick v West Norfolk and Wisbech AHA* [1985] UKHL 7.

^61^J.S.C. v. Wren, 1986 ABCA 249 (CanLII).

^62^*Freedom of Information and Protection of Privacy Regulation*, BC Reg 155/2012, 2012, s3(1).

^63^MyHealthPortal Available at: https://www.interiorhealth.ca/YourHealth/MyHealthPortal/Pages/default.aspx [Accessed February 14, 2020].

^64^*Calgary Health Region (Re)*, 2006 CanLII 80851 (AB OIPC).

^65^*Health Information Act*, RSA 2000, c H-5, s104(1)(c).

^66^*Calgary Health Region (Re)*, 2006 CanLII 80851 (AB OIPC), para 74.

^67^*Freedom of Information and Protection of Privacy Act*, RSA 2000, c F-25, s84(1)(e).

^68^*Ontario (Community Safety and Correctional Services) (Re)*, 2016 CanLII 25549 (ON IPC).

^69^General, O Reg 329/04 (*Personal Health Information Protection Act*, 2004), 24(1)(2).

^70^General, O Reg 114/94 (*Medicine Act*, 1991).

^71^Hospital Management, RRO 1990, Reg 965 (*Public Hospitals Act*).
