# Corrigendum: Parental Access to Children's Raw Genomic Data in Canada: Legal Rights and Professional Responsibility

**DOI:** 10.3389/fgene.2021.725850

**Published:** 2021-07-02

**Authors:** Michael J. S. Beauvais, Adrian M. Thorogood, Michael J. Szego, Karine Sénécal, Ma'n H. Zawati, Bartha Maria Knoppers

**Affiliations:** ^1^Centre of Genomics and Policy, Faculty of Medicine, McGill University, Montreal, QC, Canada; ^2^ELIXIR-LU, Luxembourg Centre for Systems Biomedicine, University of Luxembourg, Belvaux, Luxembourg; ^3^Centre for Clinical Ethics, Unity Health, Toronto, ON, Canada; ^4^Departments of Family and Community Medicine and Molecular Genetics, Dalla Lana School of Public Health, University of Toronto, Toronto, ON, Canada; ^5^Independent Researcher, Montreal, QC, Canada

**Keywords:** whole genome sequencing, personal genomics, pediatrics, privacy, individual access, ethics, consent

In the published article there was an error. An author name was incorrectly spelled as **M'an H. Zawati** instead of **Ma'n H. Zawati**.

The authors apologize for this error and state that this does not change the scientific conclusions of the article in any way. The original article has been updated.

